# Simultaneous precise editing of multiple genes in human cells

**DOI:** 10.1093/nar/gkz669

**Published:** 2019-08-08

**Authors:** Stephan Riesenberg, Manjusha Chintalapati, Dominik Macak, Philipp Kanis, Tomislav Maricic, Svante Pääbo

**Affiliations:** Department of Evolutionary Genetics, Max Planck Institute for Evolutionary Anthropology

## Abstract

When double-strand breaks are introduced in a genome by CRISPR they are repaired either by non-homologous end joining (NHEJ), which often results in insertions or deletions (indels), or by homology-directed repair (HDR), which allows precise nucleotide substitutions to be introduced if a donor oligonucleotide is provided. Because NHEJ is more efficient than HDR, the frequency with which precise genome editing can be achieved is so low that simultaneous editing of more than one gene has hitherto not been possible. Here, we introduced a mutation in the human *PRKDC* gene that eliminates the kinase activity of the DNA-dependent protein kinase catalytic subunit (DNA-PKcs). This results in an increase in HDR irrespective of cell type and CRISPR enzyme used, sometimes allowing 87% of chromosomes in a population of cells to be precisely edited. It also allows for precise editing of up to four genes simultaneously (8 chromosomes) in the same cell. Transient inhibition of DNA-PKcs by the kinase inhibitor M3814 is similarly able to enhance precise genome editing.

## INTRODUCTION

When CRISPR nucleases and guide RNAs (gRNAs) are used to introduce double-stranded breaks (DSBs) in a genome, the two DNA ends are covered by DNA-dependent protein kinase (DNA-PK) complexes, which consist of Ku70/80 and DNA-PKcs (1). DNA-PKcs then undergoes conformational changes induced in part by autophosphorylation (2,3) as well as by other kinases (4,5) at >60 phosphorylation sites (6–8). This allows it to recruit NHEJ repair proteins and inhibit HDR proteins, for example ataxia telangiectasia mutated kinase (ATM), by phosphorylation (6,9,10). DNA ends can also be bound by the MRN (Mre11, Rad50 and Nbs1) complex, which results in 5′-3′ resection of DNA ends and HDR or microhomology-mediated end joining (MMEJ), also referred to as alternative NHEJ (11,12). In the case of HDR, end resection is followed by RAD51-nucleoprotein filament generation, annealing of homologous DNA, and DNA synthesis (1,13). In case of MMEJ repair, identical sequences of 1–16 nucleotide in the single-stranded ends align to each other, followed by removal of unaligned parts of the single-stranded ends, gap filling DNA synthesis and ligation, resulting in a deletion at the DSB site (12).

Because NHEJ introduce indels that can lead to frameshift mutations it is often used to inactivate genes. In contrast, HDR normally uses a sister chromatid to repair the chromosome carrying a DNA break. If exogenous ‘donor’ DNA carrying desired mutations is provided, it is possible to perform precise genome editing (PGE), i.e. introduce desired nucleotide substitutions at specific positions in a genome. Typically, NHEJ is more efficient than HDR and has been used to knock out multiple target genes in cells, for example five genes in mouse embryonic stem cells (14) and 62 copies of one endogenous retrovirus in a porcine cell line (15). In contrast, although several studies have tried to inhibit NHEJ or enhance HDR in order to increase the efficiencies of PGE (16–20), simultaneous PGE of more than one desired target in animal cells has not been reported, although methods for co-selection of one desired edit by simultaneous introduction of a selectable modification at a different locus have been developed (21,22). However, this allows only a single desired target to be edited together with the selectable marker.

Because simultaneous knockouts of several genes by NHEJ is possible, DNA cleavage by the CRISPR enzymes is not rate limiting for genome editing. Rather, it seems that the higher efficiency of NHEJ relative to HDR limits the ability to introduce precise edits. In 2008, it was shown that a lysine to arginine mutation at position 3753 near the ATP binding site in the DNA-PKcs protein (KR) abolishes its kinase activity (13,23) in Chinese hamster ovary cells and that this increases homologous recombination to levels 2- to 3-fold above those seen when DNA-PKcs was completely knocked down (23). Inactive but structurally intact DNA-PKcs may increase homologous recombination because phosphorylation of downstream NHEJ proteins as well as phosphorylation-induced inhibition of ATM kinase activity (9) is blocked, while ATM levels needed for efficient HDR are maintained (23). In contrast, in the absence of DNA-PKcs protein, levels of ATM and thus HDR are reduced (13,24). Knock down of the DNA-PKcs protein by siRNA (25), or in DNA-PKcs (-/-) cell line (26), could only moderately increase homologous recombination efficiencies.

## METHODS

### Cell culture

We recently created an iCRISPR-Cas9n line from human induced pluripotent stem cells (hiPSCs) (17) (409-B2, female, Riken BioResource Center) as described by Gonzalez *et al.* (17,27) (GMO permit AZ 54-8452/26). For this study, we further used HEK293 cells (ECACC, 85120602) with Dulbecco's modified Eagle's medium/F-12 (Gibco, 31330-038) supplemented with 10% fetal bovine serum (FBS) (SIGMA, F2442) and 1% NEAA (SIGMA, M7145); as well as K562 cells (ECACC, 89121407) with Iscove's modified Dulbecco's media (ThermoFisher, 12440053) supplemented with 10% FBS. 409-B2 hiPSCs were grown on Matrigel Matrix (Corning, 35248) in mTeSR1 medium (StemCell Technologies, 05851) with supplement (StemCell Technologies, 05852) that was replaced daily. Medium for HEK293 and K562 was replaced every second day. At ∼80% confluency, adherent cells were dissociated using EDTA (VWR, 437012C) and split 1:6 to 1:10 in medium supplemented with 10 μM Rho-associated protein kinase (ROCK) inhibitor Y-27632 (Calbiochem, 688000) for 1 day after replating. K562 cells were split 1:6 to 1:10 dilution after 1 week. All cells were grown at 37°C in a humidified incubator with 5% CO_2_.

### Lipofection of oligonucleotides

**Figure 1.**
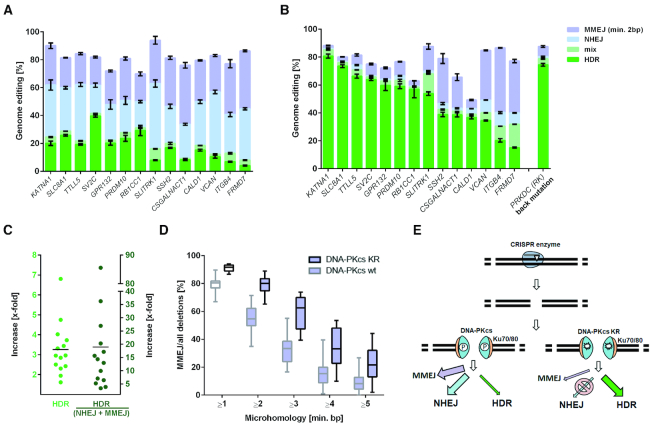
Catalytically inactive DNA-PKcs promotes homology-directed repair (HDR). (**A**) The genome editing frequencies with Cas9n and single stranded DNA donors for 14 genes in the DNA-PKcs WT cell line; and (**B**) in the DNA-PKcs KR cell line. HDR, ‘mix’ (HDR with indels), NHEJ, and MMEJ (≥2 bp microhomology) are indicated in green, light green, light blue and light purple, respectively. Error bars show the SEM of at least four replicate experiments. (**C**) Increase in frequency of HDR (left) and relative to NHEJ and MMEJ (right). Each dot corresponds to one gene and the black line shows the mean. (**D**) Percent of all deletions resulting from MMEJ for the 14 genes as a function of microhomology length in KR and WT cells. Boxes extend from the 25th to 75th percentile, show the median as a line and have whiskers from minimum to maximum values. (**E**) Schematic illustration of the repair of DSBs. After a DSB induced, e.g. by a CRISPR enzyme, DNA ends are covered by Ku70/80 (orange), followed by binding of DNA-PKcs (cyan blue), both constituting a DNA-PK complex. Autophosphorylation of DNA-PKcs leads to recruitment and activation of downstream NHEJ proteins. If DNA-PKcs is catalytically inactivated (e.g. by the K3753R mutation), the NHEJ pathway is blocked and only MMEJ can compete with HDR.

409-B2 iCRISPR-Cas9n hiPSCs were incubated in medium containing 2 μg/ml doxycycline (Clontech, 631311) three to 4 days prior to lipofection. Lipofection was done using the alt-CRISPR protocol (IDT) at a final concentration of 7.5 nM of each gRNA (crRNA/tracR duplex) and 10 nM of the DNA donors. In brief, 0.75 μl RNAiMAX (Invitrogen, 13778075) and the respective oligonucleotides were separately diluted in 25 μl OPTI-MEM (Gibco, 1985-062) and incubated at room temperature for 5 min. Both dilutions were mixed to yield 50 μl of OPTI-MEM including RNAiMAX, gRNAs and single stranded DNA donors (ssODNs). The lipofection mix was incubated for 20–30 min at room temperature. Cells were dissociated using EDTA for 5min and counted using the Countess Automated Cell Counter (Invitrogen). The lipofection mix, 100 μl containing 25 000 dissociated cells in mTeSR1 supplemented with Y-27632, and 2 μg/ml doxycycline were put in one well of a 96-well covered with Matrigel Matrix (Corning, 35248). Media was exchanged to regular mTeSR1 media after 24 h. *KATNA1, SLITRK1*, and *CALD1* were edited using lipofection (Figure 1A and B). We also attempted multiplexed editing of three genes simultaneously using lipofection, but achieved 3–10% HDR for a gene at best.

### Oligonucleotide and ribonucleoprotein electroporation

The recombinant *Streptococcus pyogenes* Cas9 protein, *Acidaminococcus* sp. *BV3L6* Cpf1 protein and respective electroporation enhancer were from IDT (Coralville, USA) and electroporation was done using the manufacturer's protocol, except for the following alterations. Nucleofection was done using the B-16 program of the Nucleofector 2b Device (Lonza) in cuvettes for 100 μl Human Stem Cell nucleofection buffer (Lonza, VVPH-5022), containing 1 million cells, 78pmol electroporation enhancer, 160 pmol of each gRNA (crRNA/tracR duplex for Cas9 and crRNA for Cpf1), 200 pmol of each single stranded DNA donor (ssODN), and 252 pmol Cas9 or Cpf1. Cells were counted using the Countess Automated Cell Counter (Invitrogen). For multiplexing, only gRNAs and ssODNs were electroporated, since a Cas9n expressing iCRISPR-Cas9n hiPSC line was used. 409-B2 iCRISPR-Cas9n hiPSCs were incubated in medium containing 2 μg/ml doxycycline (Clontech, 631311) three to four days prior to editing. For multiplexed precise genome editing, this medium was exchanged to StemFlex with supplement (Gibco, A3349401), CloneR (StemCell Technologies, 05888), and doxycycline one day before electroporation. 90% of the electroporated cells were plated for bulk genotype analysis and for hiPSCs 10 percent were plated in a separate 6well to give rise to colonies derived from a single cell (clones) for which the media was supplemented with ROCK inhibitor Y-27632 for 2 days post-electroporation. After at least 7 days colonies were picked for following propagation and DNA isolation. Single cell colonies of HEK293 and K562 cells were acquired by sorting single cells in wells of a 96-well plate using a single-cell printer (Cytena).

### Illumina library preparation and sequencing

At least 3 days after transfection cells were dissociated using Accutase (SIGMA, A6964), pelleted, and resuspended in 15 μl QuickExtract (Epicentre, QE0905T). Incubation at 65°C for 10 min, 68°C for 5 min and finally 98°C for 5 min was performed to yield single stranded DNA as a PCR template. Primers for each targeted loci containing adapters for Illumina sequencing were from IDT (Coralville, USA) (see Supplementary Table S1). PCR was done in a T100 Thermal Cycler (Bio-Rad) using the KAPA2G Robust PCR Kit (SIGMA, KK5024) with supplied buffer B and 3 μl of cell extract in a total volume of 25 μl. The thermal cycling profile of the PCR was: 95°C 3 min; 34× (95°C 15 s, 65°C 15 s, 72°C 15 s); 72°C 60 s. P5 and P7 Illumina adapters with sample specific indices were added in a second PCR reaction (28) using Phusion HF MasterMix (Thermo Scientific, F-531L) and 0.3 μl of the first PCR product. The thermal cycling profile of the PCR was: 98°C 30 s; 25× (98°C 10 s, 58°C, 10 s, 72°C 20 s); 72°C 5 min. Amplifications were verified by size separating agarose gel electrophoresis using 2% EX gels (Invitrogen, G4010–11). The indexed amplicons were purified using Solid Phase Reversible Immobilization (SPRI) beads in a 1:1 ratio of beads to PCR solution (29). Double-indexed libraries were sequenced on a MiSeq (Illumina) giving paired-end sequences of 2 × 150 bp (+7 bp index). After base calling using Bustard (Illumina) adapters were trimmed using leeHom (30).

### Amplicon sequence analysis

**Figure 2.**
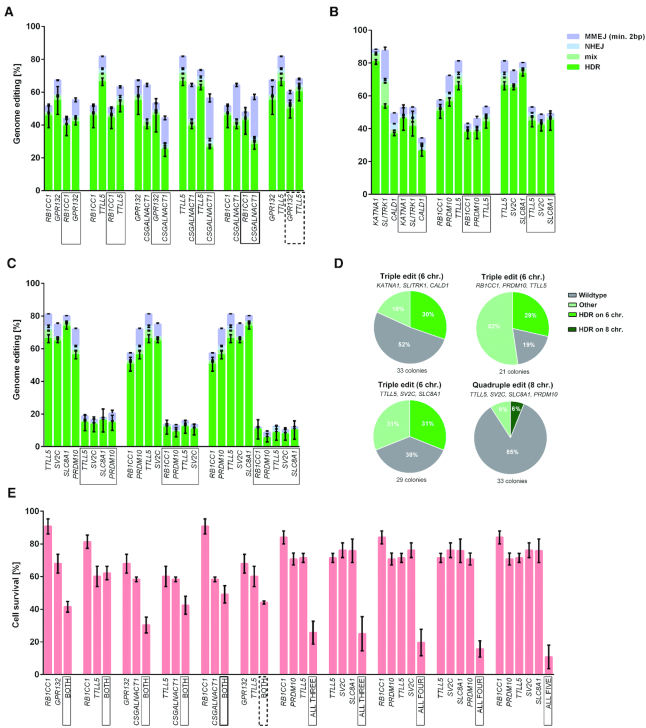
Multiplexed precise genome editing using catalytically inactive DNA-PKcs. (**A**) Editing frequencies for single genes and combinations of two out of four genes (framed). HDR, mix (HDR with indels), NHEJ, and MMEJ are indicated in green, light green, light blue and light purple, respectively. (**B**) Editing frequencies for single genes and combinations of three genes (framed). (**C**) Editing frequencies for single genes and combinations of four or five genes (framed). (**D**) Pie charts of the genotypes from single cell derived colonies after multiplexed editing of three combinations of three genes and one of four genes. See Supplementary Tables S2–S5 for details. (**E**) Cell survival after editing of one to five genes. Resazurin assays were performed three days after editing. Error bars show the SEM of four replicate experiments for A, B, C and D.The bold frame and bold dashed frame indicate that the targeted genes are on the same chromosome and are divided by a centromere (distance 34Mb) or are on the same chromosome arm (distance 29 Mb), respectively.

Bam-files were demultiplexed and converted into fastq files using SAMtools (31). CRISPResso (32) was used to analyse fastq files for percentage of wildtype, any targeted nucleotide substitution (HDR), indels (called as non-homologous end joining NHEJ), and mix of both. Analysis was restricted to amplicons with a minimum of 70% similarity to the wildtype sequence and to a window of 20 bp from each gRNA. Sequence homology for an HDR occurrence was set to 95%. Unexpected substitutions were ignored as putative sequencing errors. Since CRISPResso cannot distinguish reads with indels from NHEJ or microhomology-mediated end joining (MMEJ), we wrote a python script to call MMEJ events. Sequencing data from colonies derived from single cell seeding (Figure 2D) was analyzed using SAMtools and colonies were regarded as clones if the clear majority of reads consisted of a single sequence (homozygous) or of two sequences of similar read counts (heterozygous) and blocking mutations, ancient mutations, as well as indels for each chromosome of the cells of a clone were noted (Supplementary Tables S2–S5).

### Whole genome sequencing

**Figure 3.**
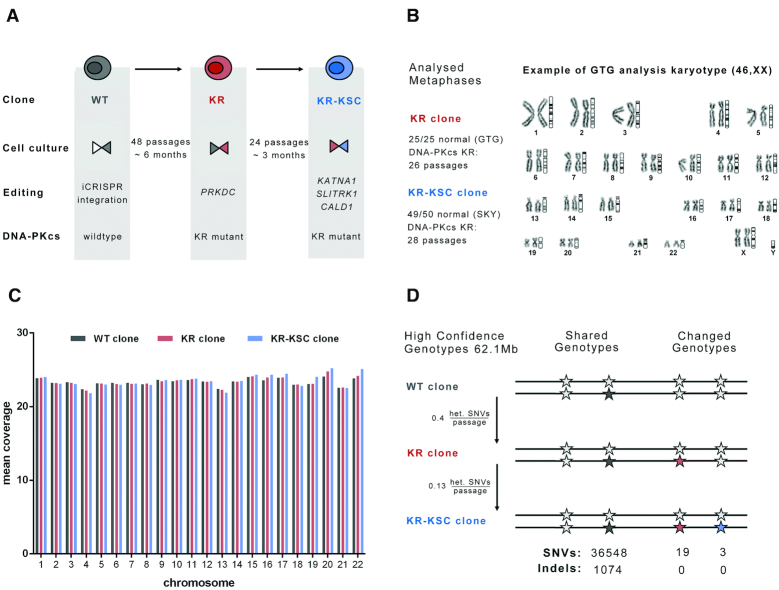
Genome stability of DNA-PKcs K3753R cells. (**A**) Schematic illustration of the cell lines used. A 409B2-iCRISPR iPSC line (gray) was edited to express the DNA-PKcs KR (red) which was in turn used to edit three genes *KATNA1, SLITRK1*, and *CALD1* to yield the KR-KSC line (blue). The editing scheme and passaging times between clonal bottlenecks are shown. (**B**) Karyotypes analyzed by Giemsa staining (GTG) spectral karyotyping (SKY) passage numbers are indicated. One polyploid metaphase was observed in the KR-KCS clone. (**C**) Genomic sequence coverage per chromosome in the three cell lines. (**D**) Numbers of SNVs and indels in high-confidence genotypes (62.1Mb) of the three cell lines are given. Novel genotypes that appeared first in the KR line (19) and KR-KSC (3) line, are colored red or blue, respectively.

The 409-B2 iCRISPR-Cas9n hiPSCs WT line, KR clone and KR-KSC line were expanded and two million cells of the respective lines were harvested and DNA was isolated. DNA was sonicated three times with a Bioruptor (Diogenode), with the output selector switched to (H)igh to yield fragments of ∼0.15 to 0.8 kb. Shearing was checked by agarose gel electrophoresis using EX gels (Invitrogen, G4010–11) and a Typhoon 9410 imager (Amersham, Biosciences). Fragment ends were made blunt for 30 min at room temperature with the Quick Blunting Kit (New Engand Biolabs, E1201L), purified with SPRI beads (28), adapter-ligated for 30 min at room temperature with the Quick Ligation Kit (New England Biolabs, M2200L), purified with SPRI beads, double indexed by PCR-amplification and purified with SPRI beads. Double-indexed libraries were sequenced on a HiSeq (Illumina) 2 × 75 bp (+7 bp index) (Figure 3C and Supplementary Figure S3).

### Mapping and genotyping

Reads were mapped to the human reference (hg19) using Burrows-Wheeler Aligner (BWA) (33) default parameters. Genotyping was carried out using Genome Analysis Toolkit (GATK) (34) (version 3.3-0-g37228af). We indel realigned the BWA mapped bam files using GATK IndelRealigner with default parameters. The variant calling was carried out on the realigned bam files by GATK HaplotypeCaller (java -jar GenomeAnalysisTK.jar -T HaplotypeCaller -R human_reference.fa -L chromosome_number –out output.vcf –I realigned.bam –emitRefConfidence BP_RESOLUTION –allSitePLs –output_mode EMIT_ALL_CONFIDENT_SITES).

### Genomic coverage calculation

Coverage along the chromosomes was calculated with for 1Mb windows, using unique genomic regions (wgEncodeDukeMapabilityUniqueness35bp=1) and excluding blacklisted regions (wgEncodeDacMapabilityConsensusExcludable, wgEncodeDukeMapabilityRegionsExcludable) (35).

### Genotype variant filtering

The variants called by GATK were filtered by removing positions that overlap repeats annotated by the UCSC genome browser and that might lead to ambiguous alignments (map35_100% filter from reference (36)). Genomic positions with 30–50-fold coverage in all three lines were used for downstream analysis. We required the absolute difference between the maximum and minimum alternate allele frequency of a genotype in all three clones to be at least 30%. Finally, we inspected each genotype differences between lines using the Integrated Genome Viewer (IGV) (37) and excluded obvious genotyping artefacts.

### Resazurin assay

409B2-iCRISPR hiPSCs expressing DNA-PKcs wildtype (WT), catalytically inactive DNA-PKcs (K3753R), or K562 cells were plated (50 000/100 000 cells per well in a 24-well plate). HiPSCs were grown for two days before treatment with different Bleomycin (Sigma, B8416) concentrations for 1 h. Afterwards cells were washed three times. After recovery for 72 h, 100 μl fresh media together with 10 μl resazurin solution (Cell Signaling, 11884) was added. Resazurin is converted into fluorescent resorfin by cellular dehydrogenases and fluorescence (exitation: 530–570 nm, emission: 590–620 nm) reflects the amount of living cells (38). Cells were incubated with resazurin at 37°C for 5 h before fluorescence readings using a Typhoon 9410 imager (Amershamn Biosciences) and quantification using ImageJ and the ‘ReadPlate’ plugin. Wells with media and resazurin, but without cells, were used a blank.

### Karyotyping

Karyotyping by trypsin induced Giemsa staining (GTG) or spectral karyotyping (SKY) were carried out according to international quality guidelines (ISCN 2016: An International System for Human Cytogenetic Nomenclature (39)) by the ‘Sächsischer Inkubator für klinische Translation’ (Leipzig, Germany).

### Cellular clone genotype and target gene copy number analysis

409-B2 hiPSCs were edited in the *FRMD7* gene using Cas9 ribonucleoprotein and DNA donor electroporation and cells were treated with or without 2 μM M3814 for 3 days. After recovery for three days cells were sorted using a single-cell printer (Cytena) with hydrophobic cartridges to establish cellular clones. DNA extracts were used for target amplification with PCR from which Illumina libraries were made and sequenced. Amplicon sequence analysis was carried out as described above. To determine *FRMD7* copy number, a TaqMan assay was done (ThermoFisher, 4400294, reporter target sequence: FAM-TTGCAGTGGGCTCTACATAGC-NFQ; human RNAse P copy number reference, ThermoFisher, 4403328; TaqMan genotyping master mix, ThermoFisher, 4371355). Quantitative PCR was carried out in a Stratagene MX3005P (Agilent Technologies). Long range PCR (∼3 kb) of the *FRMD7* locus was done in a T100 Thermal Cycler (Bio-Rad) using the KAPA2G Robust PCR Kit (SIGMA, KK5024) with buffer B and 3 μl of DNA extract in a total volume of 25 μl. The thermal cycling profile of the PCR was: 95°C 3 min; 30× (95° 15 s, 65°C 15 s, 72°C 60 s); 72°C 60 s. Primers are stated in Supplementary Table S1.

### Statistical analysis

Bar graphs in figures were plotted and SEM error bars were calculated using GraphPad Prism 6 software. The number of replicates is stated in the respective figure legends.

## RESULTS

### Catalytically inactive DNA-PKcs promotes homology-directed repair across cell types and CRISPR enzymes

To achieve high efficiency of targeted DSBs and reduce off-target DSBs we previously generated a human induced pluripotent stem cell (hiPSC) line carrying a doxycycline-inducible Cas9 with the D10A mutation (iCRISPR-Cas9n) causing it to generate nicks rather than double-stranded cuts in DNA targets (17). In this line, we now introduced the K3753R mutation in the *PRKDC* gene that encodes DNA-PKcs protein. We compared the efficiency with which nucleotide substitutions can be introduced in DNA-PKcs K3753R (KR) and DNA-PKcs wildtype (WT) cells. To do this, we designed gRNAs (40) and single-stranded oligodeoxynucleotide donors (ssODNs) to revert 14 substitutions that are fixed or almost fixed among present-day humans but occur in the ancestral, ape-like states in the Neandertal and Denisovan genomes (36) back to the ancestral states. When necessary, the ssODNs carried additional silent non-coding mutations to prevent repeated cutting of the DNA once the targeted substitutions have been introduced (Supplementary Table S1).

Following induction of expression of Cas9n by doxycycline, cells were transfected gRNAs and ssODNs and cultured for three days. DNA was isolated, PCR amplicons of the targeted regions were sequenced and HDR scored as the presence of the ssODN-derived mutations in the target genes, which could have been introduced by classical homologous recombination or single-strand template repair. When editing each gene independently, HDR frequencies in the WT cells varied between 4% and 40% among the 14 genes (average 18%). In the KR cells, frequencies varied between 15% and 81% (average 51%) (Figure 1A and B). Across the genes, HDR is increased between 1.6-fold and 6.8-fold (average 3.3-fold) in KR cells (Figure 1C).

When deletions occurred at sites where the sequence on one end of the deletion was identical to the undeleted sequence on the other end and was at least two nucleotides long we classified this as MMEJ while other indels were considered to be the result of NHEJ. When compared to DNA-PKcs WT cells, NHEJ and MMEJ decreased in KR cells from an average of 31% to 4% (8.5-fold) and 29% to 17% (1.7-fold), respectively (Figure 1A and B). The average ratio of HDR to the sum of NHEJ and MMEJ was 0.3 in WT cells and 6.5 in KR cells (Figure 1C). For all genes tested, MMEJ is increased relatively to NHEJ in KR cells, regardless of the length of microhomology involved (Figure 1D). Thus, in KR cells, NHEJ is inhibited, resulting in a drastic increase of HDR, as schematically illustrated in Figure 1E.

For the three genes with the lowest HDR frequencies in KR cells, MMEJ is the predominant editing event. To increase HDR in those genes, we tried to use Cas9 instead of Cas9n to induce chromosomal breaks. This increased HDR for two genes from 35% and 20% to 73% and 87%, respectively, while the MMEJ decreased for all three genes (Supplementary Figure S1A). Thus, for some targets where MMEJ predominates, PGE can be ‘rescued’ by using a different CRISPR enzyme, which uses different cleavage sites and therefore possibly results in different tendencies for MMEJ (Supplementary Figure S1B).

To test if the increase in HDR seen in the hiPSCs was dependent on the cell type or enzyme used, we introduced the DNA-PKcs KR mutation in human embryonic kidney cells (HEK293) and human immortalized myelogenous leukemia cells (K562). When gRNAs and ssODNs (selected from Figure 1 and a previous study (17)) are transfected together with Cas9 nickase, Cas9 or Cpf1 protein HDR increased in all cell lines regardless of the enzyme used (Supplementary Figure S1C), and regardless of if the enzyme was transfected or endogenously produced by the cell (Figure 1A, B and Supplementary Figure S1A–C).

### Simultaneous precise genome editing of multiple genes in the same cell

We next electroporated gRNAs and the corresponding donor DNAs (selected from Figure 1) for all pairwise combinations of four different genes into KR cells after induction of Cas9n expression. The HDR frequency of double edits were similar or slightly lower than single edits of the corresponding genes, regardless if both genes were located on different chromosomes, on different chromosome arms, or on the same chromosome arm (Figure 2A). We next tried to simultaneously edit three, four and five genes. For three different combinations of three genes, the HDR efficiency was on average one third lower than for the single edits leading to average editing frequencies of 40% per targeted site (Figure 2B). For four or five genes, average HDR frequencies decreased to 13% and 8%, respectively (Figure 2C). Additionally, cell survival decreased from 30–68% for two edits to 11% for five edits (Figure 2E).

We isolated single cell-derived colonies (SCCs) from the pools of edited cells. For triple edits, approximately a third of SCCs carried the edited nucleotide in a homozygous form at all genes targeted (six chromosomes) while this was the case for 6% of SCCs for quadruple edits (eight chromosomes) (Figure 2D, Supplementary Tables S2–S5). Notably, the percentage of cellular clones homozygous for all four intended amino acid-changing substitutions (6%) is 1000-fold higher than would be expected by chance if edits of the different genes would be independent of each other (0.006%), suggesting that cells that are ‘editing competent’ will tend to be efficiently edited at multiple sites.

### Genome stability of cells expressing catalytically inactive DNA-PKcs is not compromised

One concern is that inactivation of the catalytic activity of DNA-PKcs may lead to genomic instability because spontaneously occurring DSBs cannot be repaired by NHEJ. We therefore analyzed the karyotypes of hiPSCs that had expressed DNA-PKcs KR for three months by trypsin-Giemsa banding (GTG) and spectral karyotyping (SKY), which is able to detect smaller translocations. All 25 metaphases analyzed by GTG and 49 out of 50 analyzed by SKY had normal karyotypes (Figure 3B). Furthermore, we treated cells expressing either DNA-PKcs WT or KR with the DSB-inducing drug bleomycin to mimic the effect of the accumulation of DSBs over long times. As expected, bleomycin reduced cell survival. This effect was twice as strong in DNA-PKcs KR expressing cells as in WT cells (Supplementary Figure S2A). After bleomycin treatment, 4 out of 50 metaphases analyzed in DNA-PKcs WT cells contained unbalanced translocations with gain or loss of chromosomal fragments, while 2 out of 50 metaphases in DNA-PKcs KR cells contained balanced translocations (Supplementary Figure S2B–E). In conclusion, we observe no increase in aneuploidy or chromosomal rearrangements in KR cells compared to WT cells.

To asses other types of genetic instability we sequenced the genomes of three cellular clones: DNA-PKcs WT, its descendent KR clone, and KR’s descendent triple-edited KR-KSC clone (*KATNA1-SLITRK1-CALD1* edited) (Figure 3A) to ∼24-fold genomic coverage. WT cells were passaged 48 times before the KR clone was generated and KR cells were passaged 24 times before the triple-edited clone was generated (Figure 3A). Genotypes that appear in both the KR clone and the KR-KSC clone and are different from the WT clone are likely to be due to mutations that occurred while DNA-PKcs WT was expressed, whereas genotypes that appear only in the KR-KSC clone are likely to be due to mutations that happened while DNA-PKcs KR was expressed. To find big deletions and duplications we compared genomic coverage per chromosome (Figure 3C) and in 1 Mb windows along the chromosomes (Supplementary Figure S3). The only difference found was a drop in coverage in one window on chromosome 9 in both KR and KR-KSC clones compared to the WT indicating a heterozygous deletion. After filtering for high-confidence genotypes (see Methods) we looked for single nucleotide variants (SNVs) and indels among clones. Surprisingly, the telomeric 41 Mb on the short arm of chromosome 5 are devoid of heterozygote positions in the KR and KR-KSC cells whereas it contains 431 high-confidence heterozygous positions in the WT cells. This is not due to a heterozygous deletion since there is no drop in coverage and the karyotypes of chromosome 5 are normal. We speculate that this is due to a break-induced repair event, which is described in yeast and has recently been proposed to occur also in mammalian cells (41). Excluding the 41Mb on chromosome 5, we found 19 novel heterozygous SNVs shared between the KR and KR-KSC cells and three novel heterozygous SNVs in the KR-KSC cells (Figure 3D). We found no indels that differed among the three cell lines, even though we find indels shared among them. Extrapolated to the whole genome, the rate of mutation fixation per passage is 21 in WT cells and 7 in KR cells.

### Transient inactivation of DNA-PKcs by small molecule M3814

**Figure 4.**
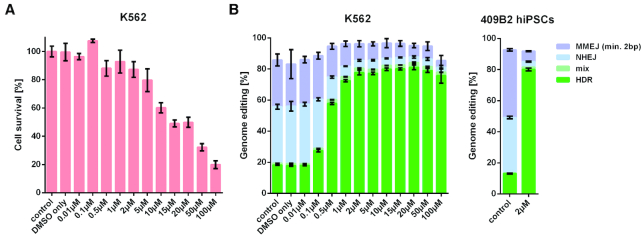
Effects of transient inactivation of DNA-PKcs by the small molecule M3814. (**A**) Resazurin assay for cell survival in K562 cells 3 days after editing of *FRMD7* with Cas9 protein and treatment with different concentrations of M3814. (**B**) Genome editing efficiencies of *FRMD7* with Cas9 protein in K562 cells treated with different concentrations of M3814, and in 409B2 hiPSCs with 2 μM M3814. M3814 was added for 3 days after editing. HDR, mix (HDR with indels), NHEJ, and MMEJ are indicated in green, light green, light blue and light purple, respectively. Error bars show the SEM of six replicates for A, three replicates for K562 cells for B, and two replicates for 409B2 hiPSCs for B.

We note that the efficiency with which nucleotide substitutions can be introduced in KR cells makes it possible to restore the normal DNA-PKcs function once desired changes have been introduced in the genome. We demonstrated this by editing the *PRKDC* gene back to its wildtype state and find that this ‘back mutation’ can be introduced in 74% of chromosomes (Figure 1B). An alternative way to increase HDR may be to transiently inhibit the kinase activity of DNA-PKcs. Indeed, several small molecule inhibitors of DNA-PK have been described to moderately increase HDR (17,25). We tested this approach using M3814 (42), a novel more efficient DNA-PKcs inhibitor which has not previously been used in genome editing. After a Cas9-induced DSB in K562 cells expressing wildtype DNA-PKcs M3814 increased HDR from 18% to 81% while exhibiting moderate toxicity. A comparable increase of HDR is seen in 409B2 hiPSCs (Figure 4A and B). The HDR increase when using M3814 is stronger than when using the previously described DNA-PK inhibitor NU7026 (4- versus 1.7-fold) (Supplementary Figure S4A).

To explore if this apparent high editing efficiency might be due to induction of large deletions of the target DNA sequences (43), we isolated cellular clones by single-cell printing after editing without and with M3814. By amplicon sequencing, we find that 10% of clones have homozygously incorporated the desired nucleotide substitution in an apparent diploid state when M3814 is not used whereas 76% have done so when M3814 is used (Supplementary Figure S5A and B). By long-range and quantitative PCR we found CRISPR-induced deletions in 2% of clones and apparent gains of one copy of the target locus in 6% of clones. The latter cases could be due to the duplication of the entire chromosome X, one of the most frequent aneuploidies in stem cells (44), or to erroneous repair of CRISPR-induced DSBs.

M3814 similarly increased HDR in several other genes and allowed for simultaneous multiple editing (data not shown). Thus, inhibition of the kinase activity of DNA-PKcs either by the reversible K3753R mutation or by the kinase inhibitor M3814 substantially increases PGE and allows the introduction of multiple nucleotide substitutions in human cells.

## DISCUSSION

The approach described here makes it possible to simultaneously edit multiple target genes in a single cell much more efficiently than is currently possible. This will facilitate the testing and potentially the correction of multiple interacting disease-related genetic variants as well as the analyses of interacting variants of physiological or evolutionary interest. In fact, PGE as described here can presumably be made even more efficient by combining it with other approaches that increase PGE, for example with combinations of small molecules (Supplementary Figure S4B and C) (17). It is likely to be applicable not only in humans but also other vertebrates because the lysine residue at position 3753 in DNA-PKcs is conserved among vertebrates (45).

It is interesting that we find no evidence that the K3753R mutation decreases genome stability. In fact, we find fewer translocations after bleomycin treatment, and fewer mutations per passage, in the mutant cells than in the DNA-PKcs WT cells. Although this could be due to unknown differences among the cell lines, it is tempting to speculate that the impairment of the error-prone NHEJ may cause the cells to more frequently repair DSBs by the less error-prone HDR. If this fails, cells may undergo apoptosis, resulting in that surviving cells maintain their genome in a more accurate form.

The fact that the DNA-PKcs inhibitor M3814 is able to increase HDR to an extent comparable to what is achievable by the *PRKDC* mutation (Figure 4) is encouraging because M3814 can be used to transiently inhibit NHEJ and thus make the modification of *PRKDC* unnecessary. It is also encouraging for work in whole organisms because permanent DNA-PKcs inactivation results in severe combined immunodeficiency in mammals due to inability to carry out V(D)J recombination (24). Indeed, M3814 is in phase Ib/II clinical trials for treatment of various cancers, for example small cell lung cancer (NCT03116971) and rectal cancer (NCT03770689). If safe in humans, M3814 could therefore find use in gene therapy applications where high HDR efficiencies may be needed to achieve therapeutic goals.

## DATA AVAILABILITY

All data are available from the corresponding author upon request. Genome sequencing data are deposited in the SRA database under accession number PRJNA543747.

## SUPPLEMENTARY DATA

Supplementary Data are available at NAR Online.

## Supplementary Material

gkz669_Supplemental_File
