# Supplementary material for: Simultaneous precise editing of multiple genes in human cells
Source: Nucleic Acids Res. 2019 Aug 8;47(19):e116. doi: 10.1093/nar/gkz669 (PMC6821318; doi:10.1093/nar/gkz669)
Supplement: gkz669_Supplemental_File [file gkz669_supplemental_file.pdf]

Supplementary Data

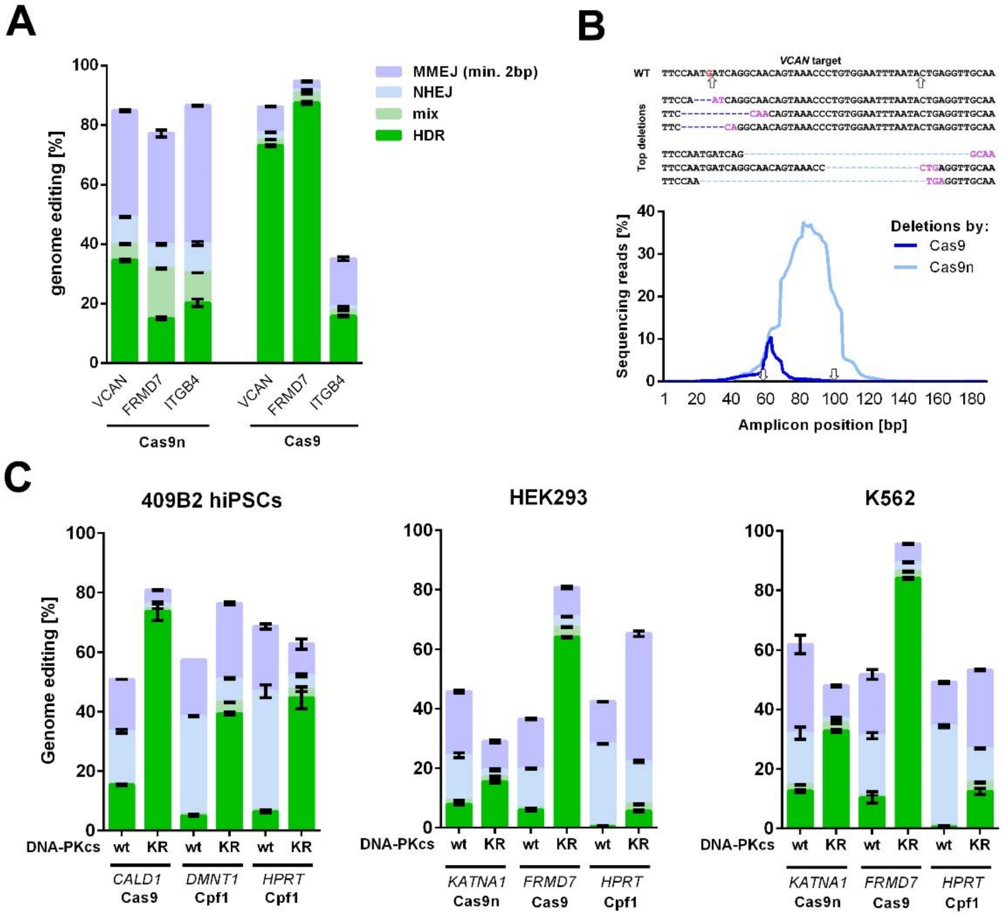

Supplementary Fig. 1. Frequency of genome editing in different DNA-PKcs K3753R cell lines using different CRISPR enzymes. (**A**) Genome editing of three genes with Cas9n (iCRISPR) and with Cas9 protein in 409B2 hiPSCs. (**B**) Patterns of deletions after editing with Cas9n and Cas9 in the gene *VCAN* as an example. Both nicking sites are indicated by arrows, the left one was used also for Cas9. The three most abundant deletions are shown for Cas9 (dark blue) and the bottom three for Cas9n (light blue). Microhomologies are indicated in pink. (**C**) The genome editing frequencies in cells expressing the DNA-PKcs wildtype (WT) or DNA-PKcs KR mutant for different recombinant CRISPR enzymes in 409B2 hiPSCs, HEK293 cells, and K562 cells. HDR, HDR with indels, NHEJ, and MMEJ are indicated in green, light green, light blue, and light purple, respectively. Error bars show the SEM of two replicates for A and four replicates for C.

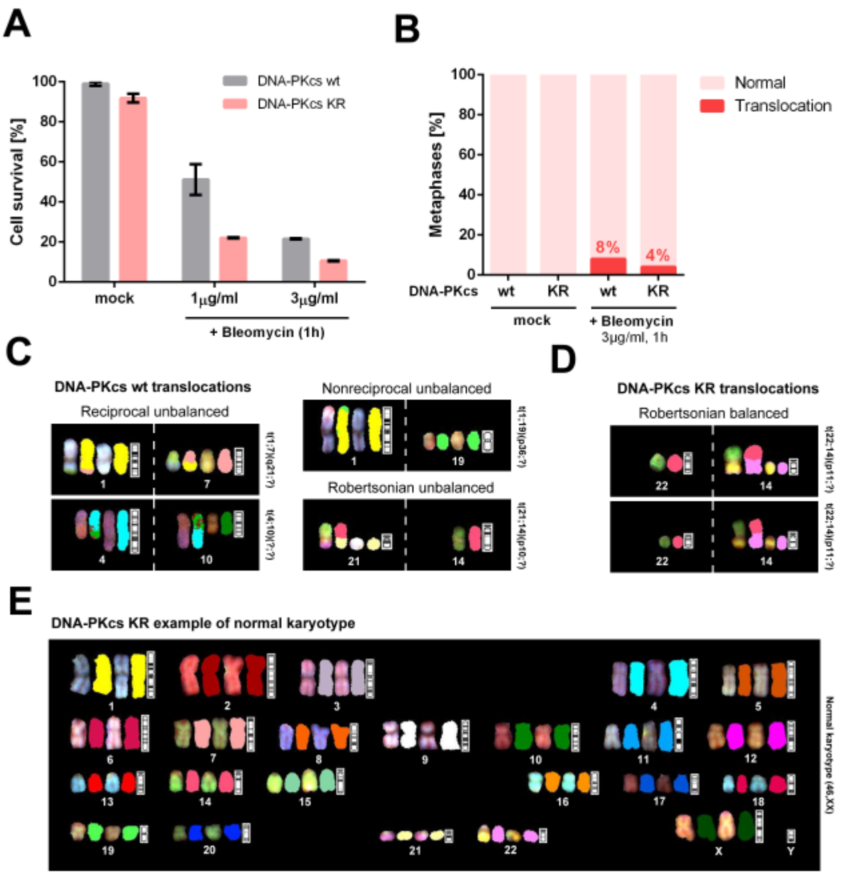

Supplementary Fig. 2. Impact of bleomycin treatment on DNA-PKcs wildtype (WT) and DNA-PKcs KR cells. (**A**) Cell survival as measured by the resazurin assay after bleomycin treatment. Error bars show the SEM of two replicates. **(B)** Spectral karyotyping (SKY) of cells after treatment with 3µg/ml bleomycin. (**C**) The translocations observed for DNA-PKcs WT cells; and **(D)** DNA-PKcs KR cells. (**E**) An example of the majority of normal metaphases observed.

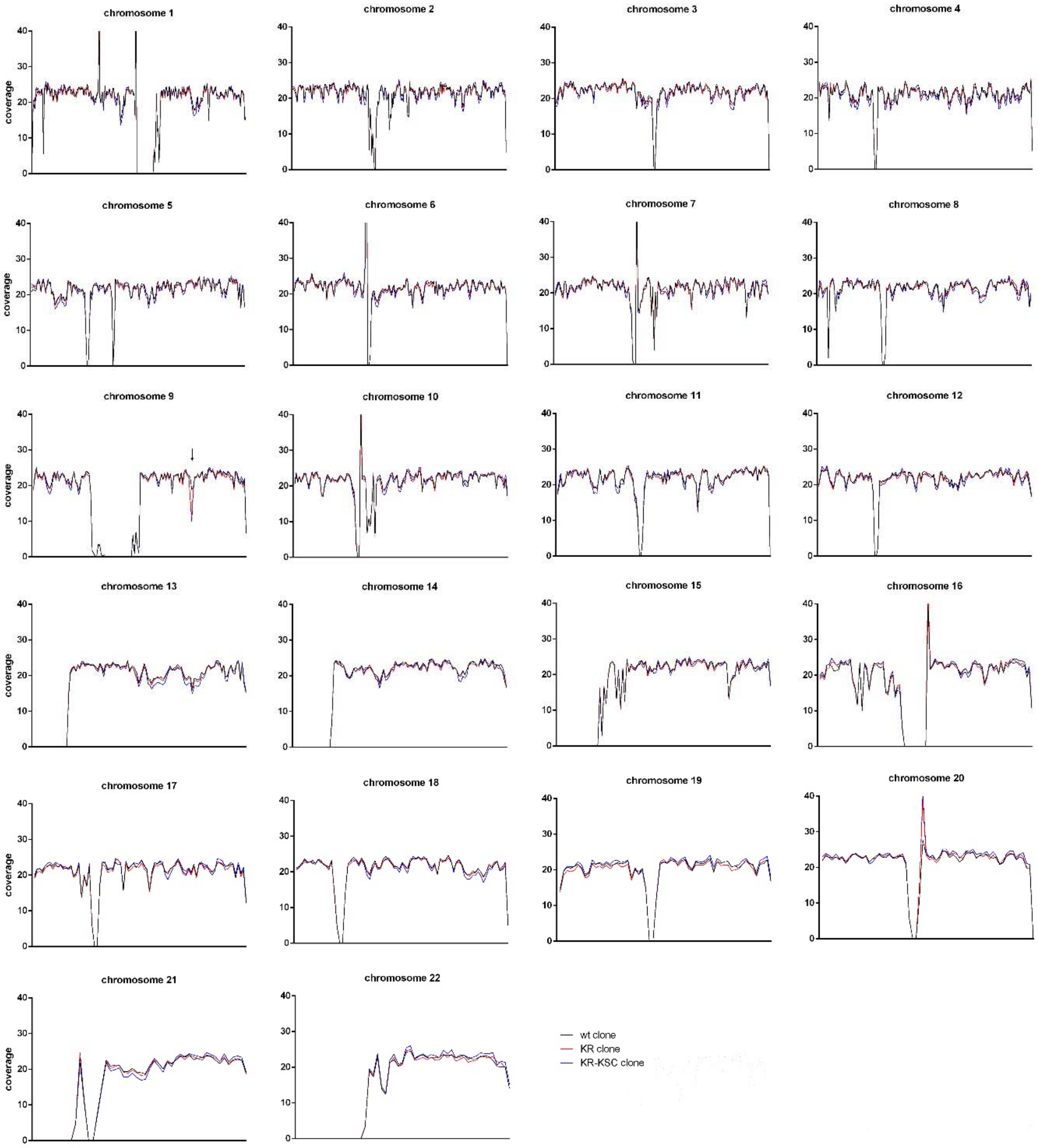

Supplementary Fig. 3. Base coverage along the chromosomes for the WT, KR, and KR-KSC hiPSC lines. Coverage was calculated for 1Mb windows, using unique genomic regions. WT, KR, and KR-KSC are indicated as a grey, red, and blue lines, respectively. A 1Mb region on chromosome 9 (arrow) carries a heterozygous deletion in the KR and KR-KSC lines.

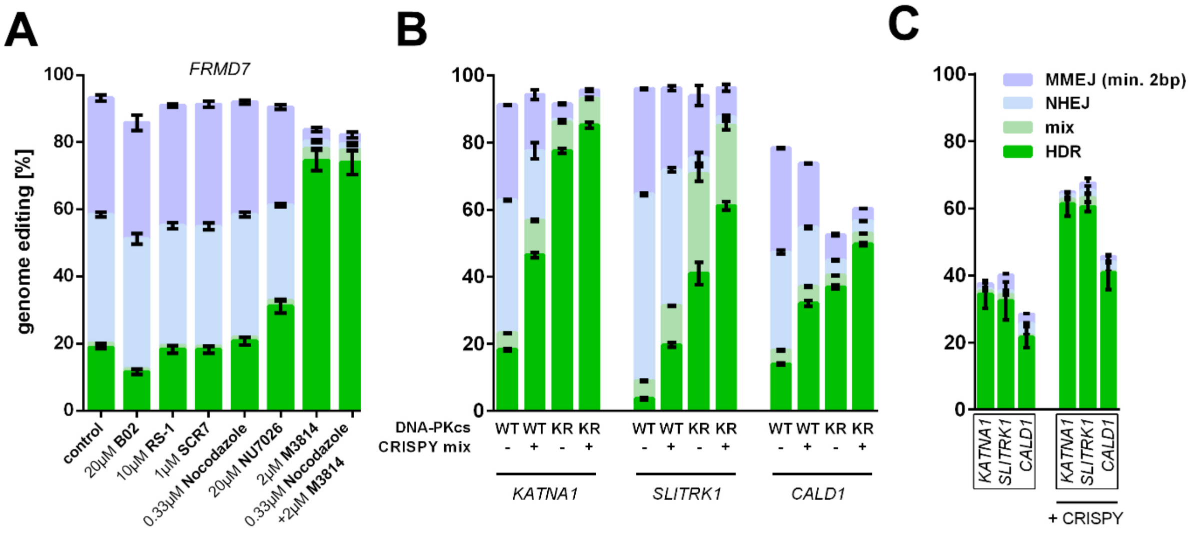

Supplementary Fig. 4. Effect of small molecules and the CRISPY mix (17) **on genome editing efficiency in 409B2 hiPSCs. (A)** Effect of RAD51 inhibitor B02, RAD51 enhancer RS-1, disputed DNA ligase IV inhibitor SCR7, cell cycle inhibitor Nocodazole (removed after 16h to achieve cell cycle synchronization), and DNA-PK inhibitors NU7026 and M3814, on genome editing of *FRMD7* with Cas9 RNP. **(B)** Genome editing of *KATNA1, SLITRK1,* and *CALD1* in DNA-PKcs WT and KR cells with or without CRISPY small molecule mix using doxycycline inducible Cas9n (iCRISPR). **(C)** Simultaneous editing of the three genes (framed) with and without the CRISPY mix. Histogram designations as in Suppl. Fig. 1. Error bars show the SEM of four replicates for A, three replicates for B and two replicates for C.

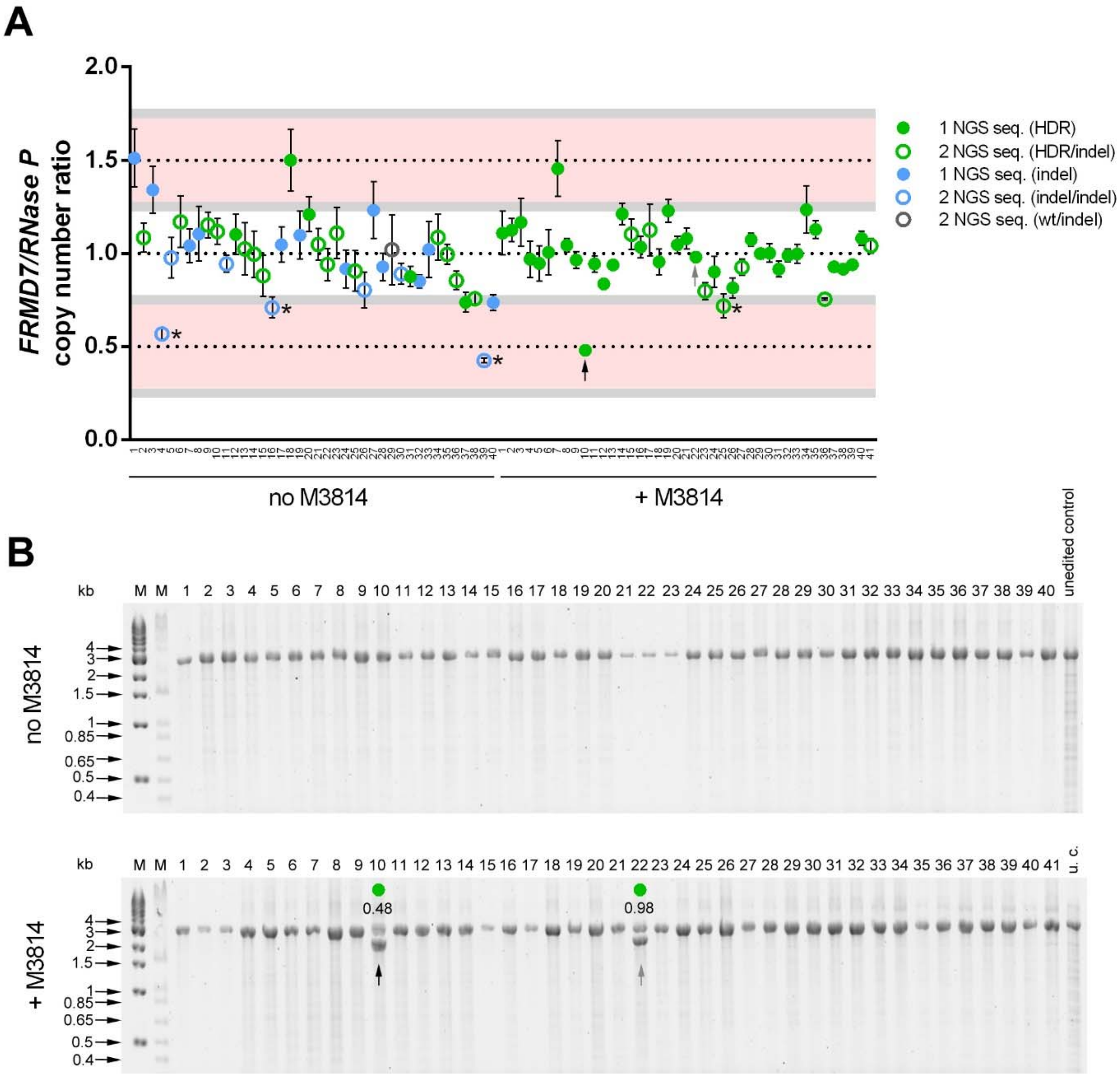

Supplementary Fig. 5 *FRMD7* gene copy number analysis in 409B2-hiPSCs edited with or without M3814. (A) Copy number ratio between *FRMD7* and the control gene *RNAse P* based on a TaqMan quantitative PCR assay. Each circle represents a cellular clone. Closed circles represent clones where amplicon sequencing yields a single DNA sequence (HDR: green; indels: blue), open circles indicate two DNA sequences of similar read number (HDR/indel mix: green; indel/indel: blue; wildtype/indel: grey). Asterisks indicate clones where a heterozygous deletion identified by DNA sequencing is expected to interfere with binding of the TaqMan probe (min. 5nt mismatch), resulting in copy number ratios below the expectation of the diploid state. Error bars show the SEM of at least four technical replicates. **(B)** Long range PCR of the *FRMD7* target locus for each clone. Arrows indicate two clones carrying heterozygous big deletions and the respective TaqMan assay copy number ratio is stated. The +M3814 clone 22 has a deletion and a copy number ratio of 0.98, suggesting a gain of one copy of the target locus in this clone as well.

Supplementary Table 1. Oligonucleotides used in this study. gRNA (gRNA 20mer target) and single stranded DNA donors (ssODNs) for editing, as well as primers for analysis are shown. Mutations are in bold letters and ancestral mutations are underlined. The gRNAs with an asterisk were used for Cas9 cleavage.

gRNAs	*KATNA1* t1	AAATGATGACCCTTCCAAAA
	KATNA1 t2	CAACACCTAAAATAAGGGTA
	SLC8A1 t1	GAGTCTCTTATTTTCCCATG
	SLC8A1 t2	CAGATGAAATCCCATTGAAA
	TTLL5 t1	CGTGGCAGGCCAGTAGGGCT
	TTLL5 t2	CGATCAGGAAGTCACACCAT
	SV2C t1	GCAGAGGTTTAATGACATCA
	SV2C t2	TGCATTGCTAACCAGAAATG
	GPR132 t1	CCATGAAGACAGACGTCACC
	GPR132 t2	TTTCCACCCCTTATGGATTC
	PRDM10 t1	TCATCACCACCACCACCAAC
	PRDM10 t2	TCTGCTGTTGGCTGTTGGTC
	RB1CC1 t1	TGAGAAAGACAGACAAAAAT
	RB1CC1 t2	CTTCGTATTTCTCTTCTTGT
	SLITRK1 t1	GCTAACAGTTTACCCTGCCC
	SLITRK1 t2	ACCCGTCGCTATCGCTGCTG
	SSH2 t1	CAGATCCTCAGGAGGGCCCA
	SSH2 t2	GTGGTCAAACTCCAGCACCT
	CSGALNACT1 t1	CTCATCTTATTTCGACCATT
	CSGALNACT1 t2	GCCGTTTGAATTCGTGTTTG
	CALD1 t1*	TGGAGACTATTGCTGCTTGA
	CALD1 t2	GCAGTATACCAGTGCAATTG
	VCAN t1*	GTTTACTGTTGCCTGATCAT
	VCAN t2	CCCTGTGGAATTTAATACTG
	ITGB4 t1	GGGTCCTGGGGTGGCAGAT
	ITGB4 t2*	CCGCAGCTGGGCAGCCGTGC
	FRMD7 t1	AGCCAGCTGAAAGAAGCCCA
	FRMD7 t2*	GTGGGCTCTACATAGCTATG
	PRKDC t1	GGTCCTCGCCACCCTTCACC
	PRKDC t2	GCGCGTGGAGCAGCTCTTCC
	PRKDC t1back	GGTCCTCGCCACCTCTCACC
	DNMT1 t1 (Cpf1)	CTGATGGTCCATGTCTGTTAC
	HPRT t1 (Cpf1)	GGTTAAAGATGGTTAAATGAT
ssODNs	*KATNA1* Cas9n	CTCATCTATATCCCAGGGAAAATTAGTAGCTGCCAGAACCATAACCATTTT**A**GAAGGGTCATCATTTTCAGAAG**C**ACCTCCAACACCTAAAATAA**C**GGTAAGGGGAGAGTGAAAAAGATATTAAGTTGGATTATACCAAATGAAGCT
	SLC8A1 Cas9n	TTCATTTCCTTCTCCTTCCATTTCTGTCTCAGCAATTACATGGTCCACATG**A**GAAAATAAGAGACTCACA**A**TAACTAACAGATGAAATCCCAT**A**GAAAAGGTGGGTGAAAGACTTAATCGCCGCATGTTGTACATGACACTTCCA
	TTLL5 Cas9n	CTGCCAGTGCTTCTCCCTGCCTACATCCCGGGGCACAGAACATCCCAAGCCCT**G**CTGGCCTGCCACGCTGTCGATCAGGAAGTCACACCATTGGTCCCTTTTCTTC
	SV2C Cas9n	ACATTTCTTTGTCTTTGCAGGTACTATGGATTATCCGTTTGGTTCCCTGA**C**GTCATTAAAC**A**TCTGCAGTCCGATGAATATGCATTGCTAACCAGAAATGTGGAGAGAGATAAAT
	GPR132 Cas9n	GCCACGGGCGACTGCAGCTCCTCGGTGTCCCTGCTGTGGGTGAGCCTGGT**A**ACGTCTGTCTTCATGGACCACT**G**TTTCCACCCCTTATG**A**ATTCTGGACACTTCTTGGCGGGAATGGTCCGTGGCCAGCACGTAGATAATG
	PRDM10 Cas9n	GGAGTCCAGGTCGAGCCACCTGCACACAGTGACTCCCTGGACCCCCAGACC**C**ACAGCCAACAGCAGACCACACAGTACATCATCACCACCACCACCAACGGGAACGGAAGCAGC
	RB1CC1 Cas9n	AAGCTTCTGAATTAACTGTTCTCTGTCTTGCTCCTGGCTGCTGACCAATTT**G**TGT**T**TGTCTTTCTCAAGGTTCTGGATAATAGCTTCGTATTTCTCTTCTTGTTGGGTAATTTTTTCATCTTTTTGTCTTTCAAGAGCACTCAATTCTGA

	SLITRK1 Cas9n	TCATCTTTAAACCCGACCCTGGGATGTGGTCGCAGCTGCAGCCCCCAGGGCA**T**GGTAAACTGTTAGCTAAGGGTTTGTTCCTGG**C**GCTACCCGTCGCTATCGC**A**GCTGTGGGTCTGATTTTGATCTGCCAGTTGCCTGGGATCTTTGTACCTCCG
	SSH2 Cas9n	ATCTGACCCTGGGCCCTCCTGAGGATCTGGCAAGTGGTCAAACTCCAGCACC**T**TGGGAGCTGGAACAGTGGCATTCTGCTCAGAATGGGACAGTGAGCCAGCCTCA
	CSGALNACT1 Cas9n	GTTGGCCATGTTGAGCTTTTCATTTTTCACTTTCATGATGGGGCCGAATGG**A**CGAAATAAGA**C**GAGCCGTTTGAATTCGTGTTTGTGGTCCCCTTTGAAGGTGAGCTCATACA
	CALD1 Cas9n	TTATATGTATGTGTTTACTTTTTTAGCAGTGGTGTCAAATCGACCCATCAAGC**T**GCA**G**TAGTCTCCAAGATTGACAGCAGACTGGAGCAGTATACCAGTGC**T**ATTGAGGTGAGAATTGTCCTCAGCGTTATGGTCCTGCTGAACAGAAATAGA
	CALD1 Cas9	GTATACTGCTCCAGTCTGCTGTCAATCTTGGAGACTA**C**TGCTGCTTGATGGGTCGATTTGACACCACTGCTAAAAAAGTAAACACATACA
	VCAN Cas9n	GATAGCAGCATCAGAACAGCAAGTGGCAGCGAGAATTCTTGATTCCAAT**A**ATCAGGCAACAGTAAACCCTGTGGAATTTAATACTGAGGTTGCAACACCAC
	VCAN Cas9	CCTGAAACTCAAGCAGCTTTAATCAGAGGGCAGGATTCCACGATAGCAGCATCAGAACAGCAAGTGGCAGCGAGAATTCTTGATTCCAAT**A**ATCAGGCAACAGTAAACCCTGTGGAATTTAATACTG
	ITGB4 Cas9n	TGGTGATGCTGCTGTACTCGCTTTGCAGCGGGTGCTGGAAGAGCCCGGCA**T**GGCTGCCCAGCTGCGGGAAGGGTCCTGGGGTGGGCAGATAGGCCAGTCAGAGGG
	ITGB4 Cas9	CTCACCCACTAGGAAGGGCTCGGTGGCGCTGGTGTGGGTGGTGGTGATGCTGCTGTACTCGCTTTGCAGCGGGTGCTGGAAGAGCCCGGCA**T**GGCTGCCCAGCTGCGGGAAGGGTCCTGGGGTGGGC
	FRMD7 Cas9n	AGGTGCCCAGATGGTCCCCAATTAGAGCAGAGGAAAGGACAAGTCCA**G**ATAGCTATGTAGAGCCCACTGCAATGAAGCCAGCTGAAAGAAGCCCAAGGAATATCAGAATG
	FRMD7 Cas9	TATGCCTCCCCAGGTCTTTTTTTATGTGGACAAGCCACCCCAGGTGCCCAGATGGTCCCCAATTAGAGCAGAGGAAAGGACAAGTCCA**G**ATAGCTATGTAGAGCCCACTGCAATGAAGCCAGCTGAA
	PRKDC Cas9n	GCGAAGGCCCAAGCGCATCATCATCCGTGGCCATGACGAGAGGGAACACCCTTTCCTGGTGA**GA**GGTGGCGAGGACCTGCGGCAGGACCAGCGCGTGGAGCAGCTCTTCCAGGTCATGAATGGGATCCTGGCCCAAG
	PRKDC back mutation Cas9n	GCGAAGGCCCAAGCGCATCATCATCCGTGGCCATGACGAGAGGGAACACCCTTTCCTGGTGA**AG**GGTGGCGAGGACCTGCGGCAGGACCAGCGCGTGGAGCAGCTCTTCCAGGTCATGAATGGGATCCTGGCCCAAG
	DNMT1 Cpf1	TTAACATCAGTACGTTAATGTTTCCTGAT**C**GTCCATGTCTGTTA**G**TCGCCTGTCAAGTGGCGTGACACCGGGCGTGTTCCCCAGAGTGAC
	HPRT Cpf1	GCCATTTCACATAAAACTCTTTTAGGTTA**T**AGATGGTTAAATGA**A**TGACAAAAAAAGTAATTCACTTACAGTCTGGCTTATATCCAACAC
**Primers**	*KATNA1* forward	CCTGACGGCAAAGGAATATAG
	KATNA1 reverse	ACTGTGCTTCCTTGTATTGTTGT
	SLC8A1 forward	AAGAAGGGTCTTGGGGTTCC
	SLC8A1 reverse	TGCCCTCTCCCCCATCTTAT
	TTLL5 forward	CCTTGCCACCATTCTTCTTT
	TTLL5 reverse	CTTTGCTGAAGAGGGACGAG
	SV2C forward	GGGAGTTGCTCATTGCCTCT
	SV2C reverse	ACCTGCCATTGTCGTATTCCA
	GPR132 forward	GCCTGGAGAAGGTGTAGTGG
	GPR132 reverse	CAGCCTCTGTGGTGTTTCTG
	PRDM10 forward	ACAGACATGAGGTGGGTGCT
	PRDM10 reverse	TCAGAATTGGAAGAAAAGCAAA
	RB1CC1 forward	GGGCAGTCTGAATAGCTTCATC
	RB1CC1 reverse	TTTGCATAACCAAGCATTTGA
	SLITRK1 forward	GGGCTTCAAATCAGCCAAG
	SLITRK1 reverse	TTTCAAGACAAATGGGCAAG
	SSH2 forward	TCAGGACTCCTTCCTGCTGT
	SSH2 reverse	GCACCAAAAGGGAAAAGTGA
	CSGALNACT1 forward	GATGCTGTCAGTGGTCAGGA
	CSGALNACT1 reverse	TCTTACCGTGCAAAGAAGGAG
	CALD1 forward	GCTAATCAGCTAGCATATGTATGAGAA
	CALD1 reverse	TTGGACTTGATTATTGTCCTAAGTG
	VCAN forward	GGCAGGATTCCACGATAGCA
	VCAN reverse	CGTGCCTTCCACTGACTCTT
	ITGB4 forward	CCATAGAGTCCCAGGATGGA
	ITGB4 reverse	GTGCTCACCCACTAGGAAGG

FRMD7 forward	TGCTCCTACCGCTAGTCCTG
FRMD7 reverse	GGTATTATGCCTCCCCAGGT
PRKDC forward	CTAGCCTGTGCCCTGAGATG
PRKDC reverse	GCACAACGCTATAGGTCCTCA
DNMT1 forward	TGAACGTTCCCTTAGCACTCTG
DNMT1 reverse	CCTTAGCAGCTTCCTCCTCC
HPRT forward	GGTGAAAAGGACCCCACGAA
HPRT reverse	TGGCAAATGTGCCTCTCTACAAAT
Illumina adapter forward 5'	ACACTCTTTCCCTACACGACGCTCTTCCGATCT
Illumina adapter reverse 5'	GTGACTGGAGTTCAGACGTGTGCTCTTCCGATCT
FRMD7 for. (long range)	AGGCCAGAACCAATCACTTC
FRMD7 rev. (long range)	GTTAGCTTCCTGGGGAGGTC
FRMD7 for. TaqMan	GGCTTCTTTCAGCTGGCTTCA
FRMD7 rev TaqMan	CCAATTAGAGCAGAGGAAAGGACAA

Supplementary Table 2. Overview of mutations introduced in *KATNA1*, *SLITRK1, and* CALD1 in 33 single cell-derived colonies (SCCs) after multiplexed editing. Integration of targeted nucleotide substitutions (left blocking mutation, 'ancient' missense mutation, and right blocking mutation) and insertion/deletions (indels) is labelled with 'y', while absence of these mutations is labelled with 'n'. Homozygous (homo) or heterozygous (het) integration of the mutations is stated. Intended substitutions or indels are highlighted with green or gray, respectively. SSC 2 has an additional unintended substitution in *CALD1* and SSC 29 has an additional unintended substitution in *KATNA1*.

	KATNA1								***SLITRK1***								***CALD1***							
	**Haplotype A**				**Haplotype B**				**Haplotype A**				**Haplotype B**				**Haplotype A**				**Haplotype B**			
SSC	Left block	Ancient	Right block	**Indel**	Left block	Ancient	Right block	**Indel**	Left block	Ancient	Right block	**Indel**	Left block	Ancient	Right block	**Indel**	Left block	Ancient	Right block	**Indel**	Left block	Ancient	Right block	**Indel**
1	y (het)	y (homo)	y (homo)	n	n	y (homo)	y (homo)	n	y (het)	n	n	n	n	y (het)	y (het)	n	y (homo)	y (homo)	n	n	y (homo)	y (homo)	n	n
2	y (homo)	y (homo)	y (homo)	n	y (homo)	y (homo)	y (homo)	n	y (homo)	y (homo)	y (homo)	n	y (homo)	y (homo)	y (homo)	n	y (homo)	y (homo)	y (homo)	n	y (homo)	y (homo)	y (homo)	n
3	n	n	n	n	n	n	n	n	n	n	n	n	n	n	n	n	n	n	n	n	n	n	n	n
4	n	n	n	n	n	n	n	n	n	n	n	n	n	n	n	n	n	n	n	n	n	n	n	n
5	n	n	n	n	n	n	n	n	n	n	n	n	n	n	n	n	n	n	n	n	n	n	n	n
6	n	n	n	n	n	n	n	n	n	n	n	n	n	n	n	n	n	n	n	n	n	n	n	n
7	y (homo)	y (homo)	y (homo)	n	y (homo)	y (homo)	y (homo)	n	y (homo)	y (homo)	y (het)	n	y (homo)	y (homo)	n	n	y (homo)	y (homo)	y (homo)	n	y (homo)	y (homo)	y (homo)	n
8	n	n	n	n	n	n	n	n	n	n	n	n	n	n	n	n	n	n	n	n	n	n	n	n
9	n	n	n	n	n	n	n	n	n	n	n	n	n	n	n	n	n	n	n	n	n	n	n	n
10	n	n	n	n	n	n	n	n	n	n	n	n	n	n	n	n	n	n	n	n	n	n	n	n
11	n	n	n	n	n	n	n	n	n	n	n	n	n	n	n	n	n	n	n	n	n	n	n	n
12	n	n	n	n	n	n	n	n	n	n	n	n	n	n	n	n	n	n	n	n	n	n	n	n
13	n	n	n	n	n	n	n	n	n	n	n	n	n	n	n	n	n	n	n	n	n	n	n	n
14	n	n	n	n	n	n	n	n	n	n	n	n	n	n	n	n	n	n	n	n	n	n	n	n
15	y (homo)	y (homo)	y (homo)	n	y (homo)	y (homo)	y (homo)	n	y (het)	y (homo)	y (homo)	y (homo)	n	y (homo)	y (homo)	y (homo)	n	n	y (homo)	n	n	n	y (homo)	n
16	n	n	n	n	n	n	n	n	n	n	n	n	n	n	n	n	n	n	n	n	n	n	n	n
17	n	n	n	n	n	n	n	n	n	n	n	n	n	n	n	n	n	n	n	n	n	n	n	n
18	y (homo)	y (homo)	y (homo)	n	y (homo)	y (homo)	y (homo)	n	y (homo)	y (homo)	y (homo)	n	y (homo)	y (homo)	y (homo)	n	y (het)	y (het)	y (homo)	n	n	n	y (homo)	n
19	y (homo)	y (het)	y (het)	n	y (homo)	n	n	y (het)	y (homo)	y (homo)	y (homo)	n	y (homo)	y (homo)	y (homo)	n	y (het)	y (het)	n	n	n	n	y (het)	n
20	n	n	n	n	n	n	n	n	n	n	n	n	n	n	n	n	n	n	n	n	n	n	n	n
21	y (homo)	y (homo)	y (homo)	n	y (homo)	y (homo)	y (homo)	n	y (het)	y (het)	y (homo)	n	n	n	y (homo)	n	y (homo)	y (homo)	y (het)	n	y (homo)	y (homo)	n	n
22	y (homo)	y (homo)	y (homo)	n	y (homo)	y (homo)	y (homo)	n	y (homo)	n	n	y (het)	y (homo)	y (het)	y (het)	n	y (homo)	y (homo)	y (het)	n	y (homo)	y (homo)	n	n
23	n	y (homo)	y (homo)	n	n	y (homo)	y (homo)	n	y (homo)	y (homo)	y (homo)	n	y (homo)	y (homo)	y (homo)	n	y (homo)	y (homo)	y (het)	n	y (homo)	y (homo)	n	n
24	n	y (homo)	y (homo)	n	n	y (homo)	y (homo)	n	y (homo)	y (homo)	y (homo)	n	y (homo)	y (homo)	y (homo)	n	y (homo)	y (homo)	y (het)	n	y (homo)	y (homo)	n	n
25	y (homo)	y (homo)	y (het)	n	y (homo)	y (homo)	n	n	y (homo)	y (homo)	y (homo)	n	y (homo)	y (homo)	y (homo)	n	y (het)	y (het)	n	n	n	n	y (het)	n
26	y (het)	y (homo)	y (homo)	y (het)	n	y (homo)	y (homo)	n	y (het)	n	n	n	n	y (het)	y (het)	n	y (het)	y (het)	n	n	n	n	y (het)	n
27	y (homo)	y (homo)	y (homo)	n	y (homo)	y (homo)	y (homo)	n	y (homo)	y (homo)	y (homo)	n	y (homo)	y (homo)	y (homo)	n	y (homo)	y (homo)	y (het)	n	y (homo)	y (homo)	n	n
28	n	n	n	n	n	n	n	n	n	n	n	n	n	n	n	n	n	n	n	n	n	n	n	n
29	y (homo)	y (homo)	y (homo)	n	y (homo)	y (homo)	y (homo)	n	y (homo)	y (homo)	y (homo)	n	y (homo)	y (homo)	y (homo)	n	y (het)	y (het)	y (homo)	n	n	n	y (homo)	n
30	n	n	n	n	n	n	n	n	n	n	n	n	n	n	n	n	n	n	n	n	n	n	n	n
31	n	n	n	n	n	n	n	n	n	n	n	n	n	n	n	n	n	n	n	n	n	n	n	n
32	y (homo)	y (homo)	y (homo)	n	y (homo)	y (homo)	y (homo)	n	y (homo)	y (het)	y (het)	n	y (homo)	n	n	n	n	n	y (homo)	n	n	n	y (homo)	n
33	y (het)	y (het)	y (het)	y (homo)	n	n	n	y (homo)	y (homo)	y (het)	y (homo)	n	y (homo)	n	y (homo)	y (het)	y (homo)	y (homo)	n	n	y (homo)	y (homo)	n	n

Supplementary Table 3. Overview of mutations introduced in *RB1CC1, PRDM10* and *TTLL5* in 21 single cell-derived colonies (SCCs) after multiplexed precise genome editing. Integration of targeted nucleotide substitutions ('ancient' missense mutation and left blocking mutation – dependent on the editing design of the respective gene) and insertion/deletions (indels) is labelled with 'y', while absence of these mutations is labelled with 'n'. Homozygous (homo) or heterozygous (het) integration of the mutations is stated. Intended substitutions or indels are highlighted with green or gray, respectively. SSC 15 has an additional unintended substitution in *RB1CC1* and *TTLL5*.

	RB1CC1						***PRMD10***				***TTLL5***			
	Haplotype A			Haplotype B			Haplotype A		Haplotype B		Haplotype A		Haplotype B	
SSC	Left block	Ancient	Indel	Left block	Ancient	Indel	Ancient	Indel	Ancient	Indel	Ancient	Indel	Ancient	Indel
1	y (het)	y (het)	n	n	n	n	y (homo)	n	y (homo)	n	y (het)	n	n	y (het)
2	y (homo)	y (homo)	n	y (homo)	y (homo)	n	y (homo)	n	y (homo)	n	y (het)	n	n	y (het)
3	y (homo)	y (het)	n	y (homo)	n	n	y (homo)	n	y (homo)	n	y (homo)	n	y (homo)	n
4	y (homo)	y (homo)	n	y (homo)	y (homo)	n	y (homo)	n	y (homo)	n	y (het)	n	n	y (het)
5	n	n	n	n	n	n	n	n	n	n	n	n	n	n
6	y (het)	y (het)	n	n	n	n	y (homo)	n	y (homo)	n	y (homo)	n	y (homo)	n
7	n	n	n	n	n	n	n	n	n	n	y (het)	n	n	n
8	n	n	n	n	n	n	y (het)	n	n	n	y (homo)	n	y (homo)	n
9	y (het)	y (het)	n	n	n	n	y (het)	n	n	n	y (homo)	n	y (homo)	n
10	n	n	n	n	n	n	y (het)	n	n	n	y (homo)	n	y (homo)	n
11	y (homo)	y (homo)	n	y (homo)	y (homo)	n	y (homo)	n	y (homo)	n	y (homo)	n	y (homo)	n
12	y (homo)	y (homo)	n	y (homo)	y (homo)	n	y (homo)	n	y (homo)	n	y (homo)	n	y (homo)	n
13	y (het)	y (het)	n	n	n	n	y (het)	n	n	y (het)	y (het)	n	n	n
14	n	n	n	n	n	n	n	n	n	n	n	n	n	n
15	y (het)	n	n	n	n	n	y (het)	n	n	y (het)	y (het)	n	n	n
16	n	n	n	n	n	n	n	n	n	y (het)	y (het)	n		n
17	y (homo)	y (homo)	n	y (homo)	y (homo)	n	y (homo)	n	y (homo)	n	y (homo)	n	y (homo)	n
18	n	n	n	n	n	n	n	n	n	n	n	n	n	n
19	y (homo)	y (homo)	n	y (homo)	y (homo)	n	y (homo)	n	y (homo)	n	y (homo)	n	y (homo)	n
20	y (homo)	y (homo)	n	y (homo)	y (homo)	n	y (homo)	n	y (homo)	n	y (homo)	n	y (homo)	n
21	n	n	n	n	n	n	n	n	n	n	n	n	n	n

Supplementary Table 4. Overview of mutations introduced in *TTLL5, SV2C,* and *SLC8A1* in 29 single cell-derived colonies (SCCs) after multiplexed precise genome editing. Integration of targeted nucleotide substitutions (‘ancient’ missense mutation, left blocking mutation, and right blocking mutation – dependent on the editing design of the respective gene) and insertion/deletions (indels) is labelled with ‘y’, while absence of these mutations is labelled with ‘n’. Homozygous (homo) or heterozygous (het) integration of the mutations is stated. Intended substitutions or indels are highlighted with green or gray, respectively. SSC 3 has an additional unintended substitution in *SLC8A1.*

	TTLL5				***SV2C***						***SLC8A1***							
	Haplotype A		Haplotype B		Haplotype A			Haplotype B			Haplotype A				Haplotype B			
SSC	Ancient	Indel	Ancient	Indel	Left block	Ancient	Indel	Left block	Ancient	Indel	Left block	Ancient	Right block	Indel	Left block	Ancient	Right block	Indel
1	y (homo)	n	y (homo)	n	y (homo)	y (homo)	n	y (homo)	y (homo)	n	y (homo)	y (homo)	n	n	y (homo)	y (homo)	n	n
2	n	n	n	n	n	n	n	n	n	n	n	n	n	n	n	n	n	n
3	n	y (het)	y (het)	n	y (homo)	y (homo)	n	y (homo)	y (homo)	n	y (homo)	y (homo)	y (homo)	y (het)	y (homo)	y (homo)	y (homo)	n
4	y (homo)	n	y (homo)	n	y (homo)	y (homo)	n	y (homo)	y (homo)	n	y (homo)	y (homo)	n	n	y (homo)	y (homo)	y (het)	n
5	n	n	n	n	n	n	n	n	n	n	n	n	n	n	n	n	n	n
6	y (homo)	n	y (homo)	n	y (homo)	y (homo)	n	y (homo)	y (homo)	n	y (homo)	y (homo)	y (homo)	n	y (homo)	y (homo)	y (homo)	n
7	n	n	n	n	n	n	n	n	n	n	n	n	n	n	n	n	n	n
8	n	n	n	n	n	n	n	n	n	n	n	n	n	n	n	n	n	n
9	n	n	n	n	n	n	n	n	n	n	n	n	n	n	n	n	n	n
10	y (homo)	n	y (homo)	n	y (homo)	y (homo)	n	y (homo)	y (homo)	n	y (het)	y (het)	y (homo)	n	n	n	y (homo)	n
11	n	n	n	n	n	n	n	n	n	n	n	n	n	n	n	n	n	n
12	y (homo)	n	y (homo)	n	y (homo)	y (homo)	n	y (homo)	y (homo)	n	y (homo)	y (homo)	y (het)	n	y (homo)	y (homo)	n	n
13	n	n	y (het)	n	n	y (homo)	n	y (het)	y (homo)	n	n	n	n	n	n	n	n	n
14	y (homo)	n	y (homo)	n	n	y (homo)	y (homo)	n	y (homo)	y (homo)	y (homo)	y (homo)	y (homo)	n	y (homo)	y (homo)	y (homo)	n
15	y (homo)	n	y (homo)	n	y (homo)	y (homo)	n	y (homo)	y (homo)	n	y (homo)	y (homo)	n	y (het)	y (homo)	y (homo)	n	n
16	n	n	n	n	n	n	n	n	n	n	n	n	n	n	n	n	n	n
17	y (homo)	n	y (homo)	n	n	n	n	n	n	n	n	n	n	n	y (het)	y (het)	n	n
18	y (homo)	n	y (homo)	n	n	n	y (het)	n	y (het)	n	n	y (homo)	y (homo)	n	n	y (homo)	y (homo)	n
19	y (homo)	n	y (homo)	n	y (homo)	y (homo)	n	y (homo)	y (homo)	n	y (het)	y (homo)	n	n	n	y (homo)	y (het)	n
20	y (homo)	n	y (homo)	n	y (homo)	y (homo)	n	y (homo)	y (homo)	n	n	y (homo)	n	n	y (het)	y (homo)	y (het)	n
21	n	n	n	n	n	n	n	n	n	n	n	n	n	n	n	n	n	n
22	n	n	n	n	n	n	n	n	n	n	n	n	n	n	n	n	n	n
23	y (homo)	n	y (homo)	n	y (homo)	y (homo)	n	y (homo)	y (homo)	n	y (homo)	y (homo)	n	n	y (homo)	y (homo)	y (het)	n
24	y (homo)	y (het)	y (homo)	n	y (homo)	y (homo)	n	y (homo)	y (homo)	n	y (homo)	y (homo)	y (het)	n	y (homo)	y (homo)	n	n
25	y (homo)	n	y (homo)	n	n	n	n	n	n	n	n	n	n	y (het)	y (het)	y (het)	n	n
26	y (homo)	n	y (homo)	n	y (homo)	y (homo)	n	y (homo)	y (homo)	n	y (homo)	n	n	n	y (homo)	y (het)	y (het)	n
27	n	n	n	n	n	n	n	n	n	n	n	n	n	n	n	n	n	n
28	n	n	n	n	n	n	n	n	n	n	n	n	n	n	n	n	n	n
29	y (homo)	n	y (homo)	n	y (homo)	n	y (het)	y (homo)	y (het)	n	y (homo)	y (homo)	n	n	y (homo)	y (homo)	y (het)	n

Supplementary Table 5. Overview of mutations introduced in *TTLL5, SV2C, SLC8A1,* and *PRDM10* in 33 single cell-derived colonies (SCCs) after multiplexed precise genome editing. Integration of targeted nucleotide substitutions ('ancient' missense mutation, left blocking mutation, and right blocking mutation – dependent on the editing design of the respective gene) and insertion/deletions (indels) is labelled with 'y', while absence of these mutations is labelled with 'n'. Homozygous (homo) or heterozygous (het) integration of the mutations is stated. Intended substitutions or indels are highlighted with green or gray, respectively.

	TTLL5				***SV2C***						***SLC8A1***								***PRMD10***			
	Haplotype A		Haplotype B		Haplotype A			Haplotype B			Haplotype A				Haplotype B				Haplotype A		Haplotype B	
SSC	Ancient	Indel	Ancient	Indel	Left block	Ancient	Indel	Left block	Ancient	Indel	Left block	Ancient	Right block	Indel	Left block	Ancient	Right block	Indel	Ancient	Indel	Ancient	Indel
1	y (homo)	n	y (homo)	n	n	n	n	n	n	n	n	y (homo)	y (homo)	n	n	y (homo)	y (homo)	n	y (het)	n	n	n
2	n	n	n	n	n	n	n	n	n	n	n	n	n	n	n	n	n	n	n	n	n	n
3	n	n	n	n	n	n	n	n	n	n	n	n	n	n	n	n	n	n	n	n	n	n
4	y (homo)	n	y (homo)	n	y (homo)	y (homo)	n	y (homo)	y (homo)	n	n	y (homo)	y (homo)	n	n	y (homo)	y (homo)	n	y (homo)	n	y (homo)	n
5	n	n	n	n	n	n	n	n	n	n	n	n	n	n	n	n	n	n	n	n	n	n
6	n	n	n	n	n	n	n	n	n	n	n	n	n	n	n	n	n	n	n	n	n	n
7	n	n	n	n	n	n	n	n	n	n	n	n	n	n	n	n	n	n	n	n	n	n
8	n	n	n	n	n	n	n	n	n	n	n	n	n	n	n	n	n	n	n	n	n	n
9	n	n	n	n	n	n	n	n	n	n	n	n	n	n	n	n	n	n	n	n	n	n
10	n	n	n	n	n	n	n	n	n	n	n	n	n	n	n	n	n	n	n	n	n	n
11	n	n	n	n	n	n	n	n	n	n	n	n	n	n	n	n	n	n	n	n	n	n
12	n	n	n	n	n	n	n	n	n	n	n	n	n	n	n	n	n	n	n	n	n	n
13	n	n	n	n	n	n	n	n	n	n	n	n	n	n	n	n	n	n	n	n	n	n
14	n	n	n	n	n	n	n	n	n	n	n	n	n	n	n	n	n	n	n	n	n	n
15	n	n	n	n	n	n	n	n	n	n	n	n	n	n	n	n	n	n	n	n	n	n
16	n	n	n	n	n	n	n	n	n	n	n	n	n	n	n	n	n	n	n	n	n	n
17	n	n	n	n	n	n	n	n	n	n	n	n	n	n	n	n	n	n	n	n	n	n
18	n	n	n	n	n	n	n	n	n	n	n	n	n	n	n	n	n	n	n	n	n	n
19	n	n	n	n	n	n	n	n	n	n	n	n	n	n	n	n	n	n	n	n	n	n
20	n	n	n	n	n	n	n	n	n	n	n	n	n	n	n	n	n	n	n	n	n	n
21	n	n	n	n	n	n	n	n	n	n	n	n	n	n	n	n	n	n	n	n	n	n
22	y (homo)	n	y (homo)	n	y (homo)	y (homo)	n	y (homo)	y (homo)	n	y (homo)	y (homo)	n	n	y (homo)	y (homo)	n	n	y (homo)	n	y (homo)	n
23	n	n	n	n	n	n	n	n	n	n	n	n	n	n	n	n	n	n	n	n	n	n
24	n	n	n	n	n	n	n	n	n	n	n	n	n	n	n	n	n	n	n	n	n	n
25	n	y (het)	n	n	n	n	n	y (het)	y (het)	n	n	n	n	n	n	n	n	n	n	n	n	n
26	n	n	n	n	n	n	n	n	n	n	n	n	n	n	n	n	n	n	n	n	n	n
27	y (homo)	n	y (homo)	n	n	n	y (het)	y (het)	y (het)	n	y (homo)	n	n	n	y (homo)	y (het)	y (het)	n	y (homo)	n	y (homo)	n
28	n	n	n	n	n	n	n	n	n	n	n	n	n	n	n	n	n	n	n	n	n	n
29	n	n	n	n	n	n	n	n	n	n	n	n	n	n	n	n	n	n	n	n	n	n
30	n	n	n	n	n	n	n	n	n	n	n	n	n	n	n	n	n	n	n	n	n	n
31	n	n	n	n	n	n	n	n	n	n	n	n	n	n	n	n	n	n	n	n	n	n
32	n	n	n	n	n	n	n	n	n	n	n	n	n	n	n	n	n	n	n	n	n	n
33	n	n	n	n	n	n	n	n	n	n	n	n	n	n	n	n	n	n	n	n	n	n
